# Caffeine in Wastewater Is a Tracer for Human Fecal Contamination

**DOI:** 10.1289/ehp.120-a108a

**Published:** 2012-03-01

**Authors:** Carol Potera

**Affiliations:** Carol Potera, based in Montana, has written for *EHP* since 1996. She also writes for *Microbe*, *Genetic Engineering News*, and the *American Journal of Nursing*.

Sewage contamination of surface waters can be a serious problem, exposing people to waterborne pathogens such as *Cryptosporidium*, *Giardia,* and norovirus via recreational waters^1^^,^^2^ and drinking water supplies.^3^ Contaminants can run off into waterways from many different sources—domestic, agricultural, and industrial—and it is not always easy to identify where contamination is coming from. A new study indicates that measuring caffeine in municipal water systems provides a good estimate of fecal contamination caused solely by humans.^4^

Researchers led by Sébastien Sauvé, an associate professor in the Department of Chemistry at the Université de Montréal, Québec, discovered that caffeine levels correlate strongly with levels of fecal coliform bacteria. Caffeine is a particularly good marker for human fecal contamination because agricultural and industrial sources of fecal coliforms generally do not release caffeine into the environment. Plus, the ubiquity of caffeine consumption means that where there is human sewage, there almost certainly will be caffeine as well.^4^

Sauvé’s team analyzed water samples collected from streams, stormwater collection pipes, and stormwater discharge points in Montréal City. They measured caffeine, fecal coliforms, and the antiseizure medication carbamazepine, another candidate chemical indicator of human sewage contamination.^5^ Of 120 samples collected, 93 exceeded 200 colony-forming units (cfu) fecal coliforms per 100 mL water.

**Figure:**
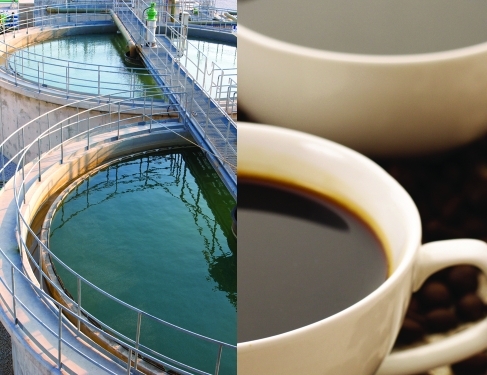
Using caffeine as a tracer to monitor fecal contamination in waterways could be a useful tool in protecting public water supplies. Shutterstock.com

Caffeine, but not carbamazepine, was strongly correlated with fecal coliform counts.^4^ All the water samples with more than 400 ng/L caffeine—an arbitrary threshold selected by the authors—were contaminated with fecal coliforms at concentrations exceeding 200 cfu/100 mL. The U.S. Environmental Protection Agency set a standard for safe swimming and recreational waters of 235 cfu/100 mL fecal coliforms,^6^ and the Canadian limit is 200 cfu/100 mL.^7^

Sauvé makes this practical comparison of his data: “Any water sample containing more than the equivalent of ten cups of coffee diluted in an Olympic-size swimming pool is definitely contaminated with fecal coliforms.” He adds that ELISA kits that detect caffeine potentially could be calibrated for fieldwork.

Environmental chemist Piero Gardinali of Florida International University in North Miami says that Sauvé’s results clearly indicate the correlation is relevant, and that a threshold of 400 ng/L caffeine could be used for environmental assessment. “Finding this link was extremely important,” says Gardinali.

People regularly consume caffeine in coffee, tea, soft drinks, chocolate, and medications,^8^ and after excretion, caffeine degrades slowly in the environment.^5^ Caffeine offers several advantages as a tracer of environmental fecal contamination. For one, it’s faster than time-consuming bacterial cultures now used to measure fecal coliforms. The presence of caffeine indicates exclusively human fecal pollution, whereas fecal coliform cultures usually cannot differentiate human excrement from that of pets, wildlife, and livestock. The discovery also offers public works officials a potential tool for locating sewage leaks. “If there’s no caffeine upstream but there’s caffeine downstream, then the sewage leak lies in between,” Sauvé says.

Sauvé’s study also showed that parts of Montréal’s stormwater collection system, which combines rain and domestic sewers, causes sewage contamination of surface waters. Ideally, uncontaminated stormwater should flow into a river, while sewage is delivered to wastewater treatment plants. But in Montréal and many other cities, stormwater runoff and sanitary sewage both run into so-called combined sewer systems that can overflow during heavy rainfalls.^9^ “Any big city where sewers and runoff combine have cross-contamination problems,” Sauvé says.
